# Supplementary material for: End Sequence Analysis Toolkit (ESAT) expands the extractable information from single-cell RNA-seq data
Source: Genome Res. 2016 Oct;26(10):1397–410. doi: 10.1101/gr.207902.116 (PMC5052061; doi:10.1101/gr.207902.116)
Supplement: Supplemental Material [file supp_gr.207902.116_Supp_Materials.tar.gz › ESAT-esat_v0.1/doc/html/classbroad_1_1pda_1_1seq_1_1rap_1_1rna_1_1_collect_rna_seq_metrics.html]

ESAT: broad.pda.seq.rap.rna.CollectRnaSeqMetrics Class Reference


ESAT


- Main Page
- Related Pages
- Packages
- Classes
- Files

- Class List
- Class Index
- Class Hierarchy
- Class Members

All Classes Namespaces Files Functions Variables Enumerator Pages

- broad
- pda
- seq
- rap
- rna
- CollectRnaSeqMetrics

Static Public Member Functions |
Public Attributes |
Protected Member Functions |
List of all members

broad.pda.seq.rap.rna.CollectRnaSeqMetrics Class Reference

Inheritance diagram for broad.pda.seq.rap.rna.CollectRnaSeqMetrics:

[legend]

Collaboration diagram for broad.pda.seq.rap.rna.CollectRnaSeqMetrics:

[legend]

|  |  |
| --- | --- |
| Static Public Member Functions | |
| static void | main (final String[] argv) |
|  | |

|  |  |
| --- | --- |
| Public Attributes | |
| final String | USAGE |
|  | |
| File | EXCLUDE\_BED =null |
|  | |
| File | EXCLUDE\_REGION\_FILE =null |
|  | |
| Integer | BUFFER = 1000 |
|  | |

|  |  |
| --- | --- |
| Protected Member Functions | |
| void | setup (final SAMFileHeader header, final File samFile) |
|  | |
| void | acceptRead (final SAMRecord rec, final ReferenceSequence refSeq) |
|  | |
| void | finish () |
|  | |

## Member Function Documentation

|  |  |  |  |
| --- | --- | --- | --- |
| void broad.pda.seq.rap.rna.CollectRnaSeqMetrics.acceptRead | ( | final SAMRecord | *rec*, |
|  |  | final ReferenceSequence | *refSeq* |
|  | ) |  |  |

protected

|  |  |  |  |  |
| --- | --- | --- | --- | --- |
| void broad.pda.seq.rap.rna.CollectRnaSeqMetrics.finish | ( |  | ) |  |

protected

|  |  |  |  |  |  |
| --- | --- | --- | --- | --- | --- |
| static void broad.pda.seq.rap.rna.CollectRnaSeqMetrics.main | ( | final String[] | *argv* | ) |  |

static

Required main method implementation.

|  |  |  |  |
| --- | --- | --- | --- |
| void broad.pda.seq.rap.rna.CollectRnaSeqMetrics.setup | ( | final SAMFileHeader | *header*, |
|  |  | final File | *samFile* |
|  | ) |  |  |

protected

## Member Data Documentation

Integer broad.pda.seq.rap.rna.CollectRnaSeqMetrics.BUFFER = 1000

File broad.pda.seq.rap.rna.CollectRnaSeqMetrics.EXCLUDE\_BED =null

File broad.pda.seq.rap.rna.CollectRnaSeqMetrics.EXCLUDE\_REGION\_FILE =null

final String broad.pda.seq.rap.rna.CollectRnaSeqMetrics.USAGE

**Initial value:**

= getStandardUsagePreamble() +

"Program to collect metrics about the alignment of RNA to various functional classes of loci in the genome:" +

" coding, intronic, UTR, intergenic, ribosomal.\n" +

"Also determines strand-specificity for strand-specific libraries.\n" +

"For RAP, provide a EXCLUDE\_REGION specifying the bounds of the target RNA. Metrics will be calculated excluding this region."

---

The documentation for this class was generated from the following file:

- java/broad/pda/seq/rap/rna/CollectRnaSeqMetrics.java


---

Generated on Fri Aug 15 2014 15:11:03 for ESAT by  

 1.8.7
